# *Mycobacterium goodii* pulmonary disease with organizing pneumonia: A case report and review of literature

**DOI:** 10.1002/rcr2.70095

**Published:** 2025-01-04

**Authors:** Yu Shionoya, Hajime Kasai, Reiya Kono, Ryutaro Hirama, Masayuki Ota, Akira Naito, Mitsuhiro Abe, Takeshi Kawasaki, Jun‐ichiro Ikeda, Takuji Suzuki

**Affiliations:** ^1^ Department of Respirology, Graduate School of Medicine Chiba University Chiba Japan; ^2^ Department of Medical Education, Graduate School of Medicine Chiba University Chiba Japan; ^3^ Health Professional Development Center Chiba University Hospital Chiba Japan; ^4^ Department of Diagnostic Pathology, Graduate School of Medicine Chiba University Chiba Japan; ^5^ Department of Respirology Japanese Red Cross Narita Hospital Chiba Japan

**Keywords:** antimycobacterial therapy, *Mycobacterium goodii*, non‐tuberculous mycobacterium, organizing pneumonia, systemic corticosteroids

## Abstract

*Mycobacterium goodii*, a rapidly growing non‐tuberculous mycobacterium, rarely causes pulmonary diseases. A patient was admitted to our hospital with a fever and cough. Chest radiography revealed consolidation in the right middle lung. As he had previously been treated for organizing pneumonia (OP), he was diagnosed with OP recurrence and administered systemic corticosteroids. Although initial improvement was observed, the pulmonary consolidations worsened. Transbronchial lung cryobiopsy revealed an OP pattern. *M. goodii* was identified in sputum acid‐fast bacilli cultures. The patient was diagnosed with *M. goodii* pulmonary disease and secondary OP. Although intravenous imipenem‐cilastatin, amikacin, and ciprofloxacin led to initial improvement in pulmonary consolidations, the consolidations re‐worsened. Systemic corticosteroids were initiated, resulting in improvement in the consolidations. The dose of systemic corticosteroids was tapered; oral antimycobacterial therapy was continued. *M. goodii* can cause pulmonary disease and induce OP; antimycobacterial therapy and systemic corticosteroids can be effective.

## Graphical abstract

This case highlights the rare instance of *Mycobacterium goodii* pulmonary disease accompanied by organizing pneumonia. The successful treatment involved a multidrug antimycobacterial therapy based on in vitro susceptibility testing, combined with systemic corticosteroids.

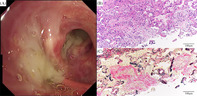

## INTRODUCTION

*Mycobacterium goodii* is a rapidly growing non‐tuberculous mycobacterium (NTM). Brown et al. have reported that *M. goodii* is a member of the *Mycobacterium smegmatis* group.^1^ *M. goodii* has been reported to be isolated from soil and water.^2^, ^3^ *M. goodii* infections occur through exposure to environmental sources and are commonly observed in patients with post‐traumatic wound infections.^1^ Only a few reports on *M. goodii* pulmonary disease have been published.^1^, ^4^, ^5^, ^6^, ^7^

Organizing pneumonia (OP) is a patchy process of lung tissue repair after injury.^8^ The OP pattern is a histological finding characterized by polypoid plugs of loose connective tissue in distal airspaces, mostly the alveoli and alveolar ducts and seldom the bronchioles.^8^ OP is usually caused by bacterial infection, drugs, or connective tissue diseases.^9^, ^10^ Although NTM pulmonary disease can induce OP, there are limited reports on such cases.^7^, ^11^, ^12^, ^13^, ^14^, ^15^, ^16^, ^17^, ^18^

The clinical features of pulmonary disease caused by *M. goodii* remain unclear. Herein, we present a case wherein *M. goodii* pulmonary disease induced secondary OP.

## CASE REPORT

**FIGURE 1:**
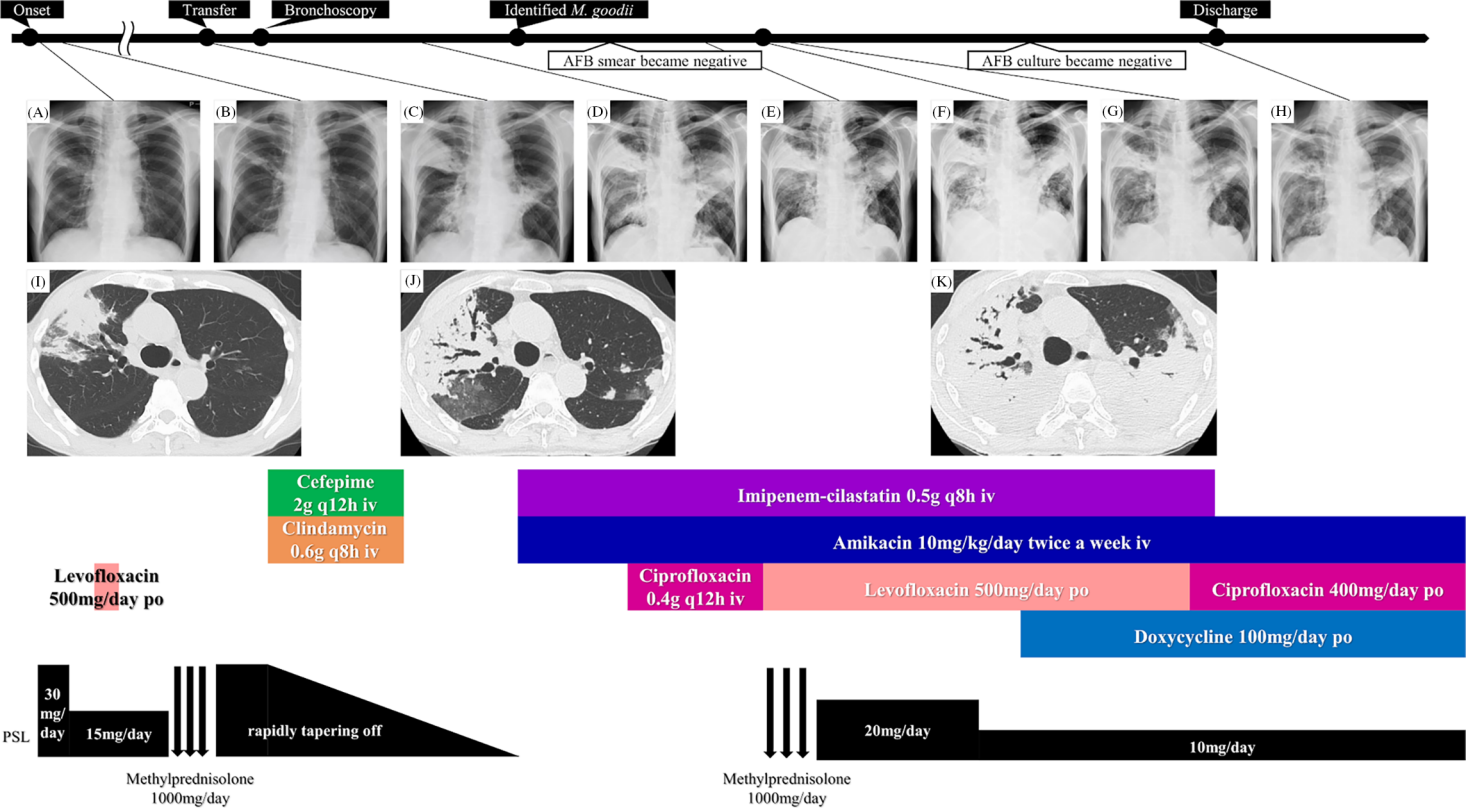
Imaging findings of chest radiography and treatments over time. (A) Chest radiography reveals consolidation in the right middle lung when symptoms developed. (B) Chest radiography reveals improvement in consolidations in the right middle lung after initiating prednisolone (30 mg/day) treatment. (C) Chest radiography reveals exacerbation of bilateral consolidations despite administration of antibiotics and methylprednisolone (1000 mg/day for 3 days). (D) Chest radiography reveals mild exacerbation of bilateral consolidations after administration of antibiotics. (E) Chest radiography reveals improvement in bilateral consolidations after initiating antimycobacterial therapy. (F) Chest radiography reveals acute exacerbation of bilateral consolidations on day 29 after hospitalization. (G) Chest radiography reveals improvement in bilateral consolidations after administration of methylprednisolone (1000 mg/day for 3 days). (H) Chest radiography reveals improvement in bilateral consolidations before discharge. (I) Chest computed tomography (CT) reveals consolidation in the right upper lobe of the lung when symptoms developed. (J) Chest CT reveals exacerbation of the consolidation in the right upper lobe of the lung and development of new consolidations in other lobes of the lung. (K) Chest CT reveals exacerbation of bilateral consolidations on day 29 after hospitalization. AFB, acid‐fast bacilli; *M. goodii*, *Mycobacterium goodii*; po, per os; PSL, prednisolone; iv, intravenous.

A man in his 80s with a history of gastrectomy visited a nearby hospital with a productive cough and fever. Chest radiography revealed consolidation in the right middle lung; chest computed tomography revealed consolidation in the right upper lung lobe (Figure 1A,I). Seven years before symptoms developed, the patient had been treated for OP; thus, he was diagnosed with OP recurrence. Therefore, oral prednisolone (30 mg/day) was administered. Although initial improvement was observed (Figure 1B), consolidations exacerbated upon prednisolone dose reduction. Despite administering antibiotics and methylprednisolone (1000 mg/day for 3 days), no improvement was observed (Figure 1C,J). Eventually, the patient developed hypoxemia and was hospitalized.

**FIGURE 2:**
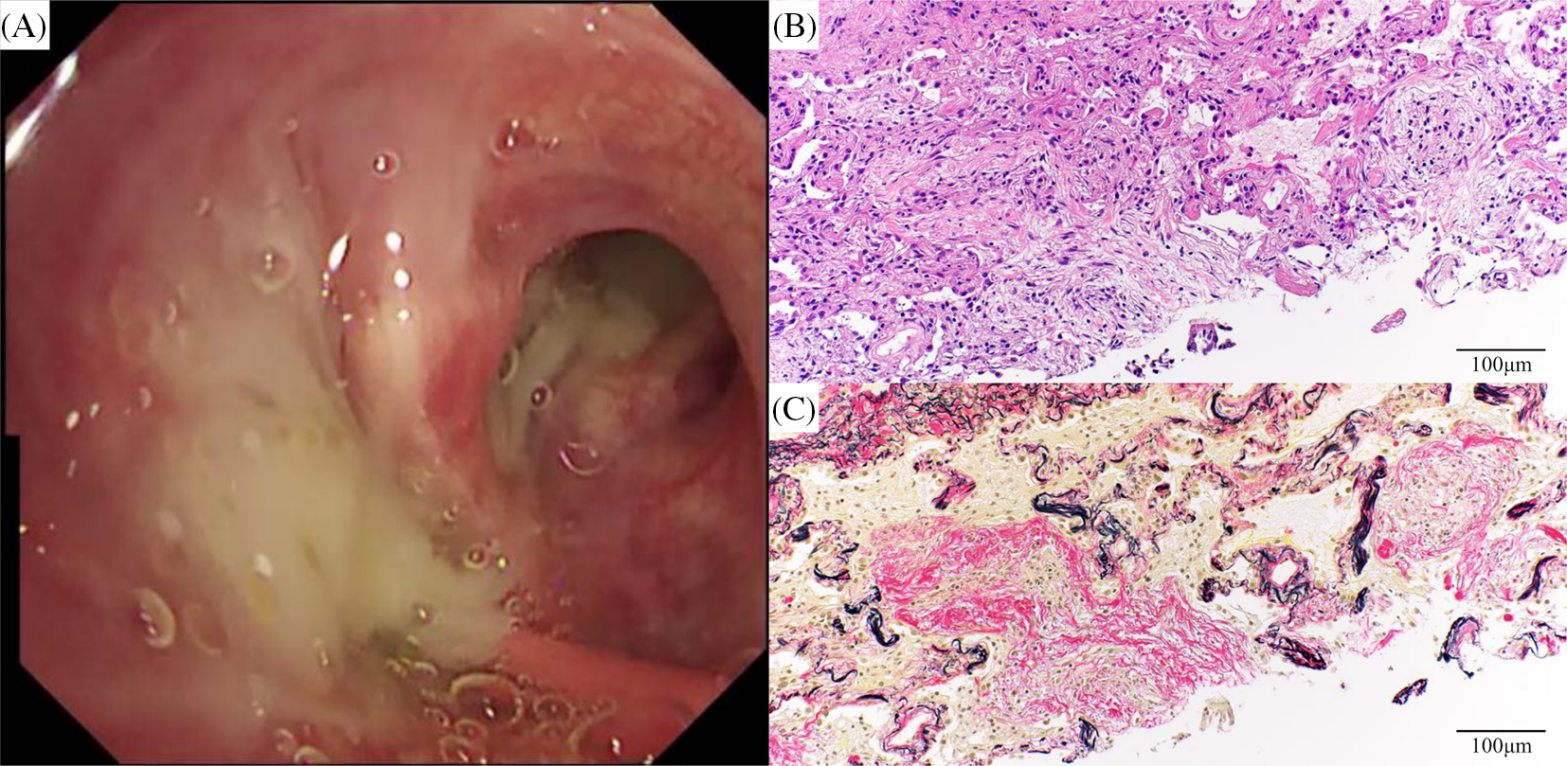
Imaging findings of bronchoscopy and pathological findings of transbronchial cryobiopsy. (A) A large amount of purulent sputum is observed in the right bronchus. (B, C) Histopathologically, lightly staining fibroblast plugs filling the alveolar spaces around the bronchi are observed, suggesting an organizing pneumonia pattern. The peribronchiolar region is not sampled, resulting in insufficient evaluation (×200 magnifications; Haematoxylin‐eosin staining and Elastica van Gieson staining).

The patient was transferred to our department for evaluation and treatment of refractory pneumonia. Upon transfer, his percutaneous oxygen saturation was 95% under oxygen administration via a nasal cannula at 2 L/min. Blood test results revealed elevated white blood cell count and C‐reactive protein levels (Table 1). No specific causes of pneumonia, such as serological autoantibodies or drugs, were identified. In the sputum culture collected before transfer from a nearby hospital, no specific bacteria were detected. Although the sputum acid‐fast bacilli (AFB) smear showed negative results, the sputum AFB culture yielded positive findings; the species were unknown. Bronchoscopy was performed on the second day after hospitalization. A large amount of purulent sputum was observed in the bronchi (Figure 2A). Transbronchial lung cryobiopsy of the right upper lung lobe revealed an OP pattern (Figure 2B,C). Analysis of bronchoalveolar lavage fluid obtained from the right middle lung lobe showed a slightly elevated lymphocyte ratio and no evidence of microbial infection, including AFB (Table 1). Based on the results of bronchoscopy, the consolidations were considered to be bacterial pneumonia and secondary OP. Despite administering antibiotics and tapering of the oral prednisolone dose, the consolidations did not improve (Figure 1D). His respiratory condition worsened, developing respiratory failure. Sputum AFB tests were repeated twice, immediately after bronchoscopy and on the day following bronchoscopy. Consecutive sputum AFB smears and cultures revealed positive results. On day 16 after hospitalization, *M. goodii* was identified in all sputum AFB cultures, obtained prior to the transfer from a nearby hospital, immediately after bronchoscopy, and on the day following bronchoscopy (Table 1). *M. goodii* were identified by analysing the homology of *rpoB*, *hsp65*, *secA1*, and *sodA* genes, using the basic local alignment search tool (BLAST). Ultimately, refractory pneumonia was diagnosed as *M. goodii* pulmonary disease and secondary OP. On the same day, intravenous amikacin (10 mg/kg/day, twice weekly) and imipenem‐cilastatin (0.5 g q8h) were administered. Additionally, intravenous ciprofloxacin (0.4 g q12h) was administered. Subsequently, respiratory failure and consolidations on chest radiography improved (Figure 1E). On day 22 after hospitalization, although the sputum AFB smear showed negative results, the culture findings remained positive. On day 29 after hospitalization, respiratory failure worsened. Chest radiography and computed tomography revealed exacerbation of the consolidations (Figure 1F,K), which was considered worsened OP. Therefore, methylprednisolone (1000 mg/day for 3 days) was administered. Furthermore, considering the complications of bacterial pneumonia, ciprofloxacin was substituted with oral levofloxacin (500 mg, q24h). Subsequently, consolidations on chest radiography and respiratory failure improved (Figure 1G). Methylprednisolone was switched to oral prednisolone (20 mg/day). Subsequently, the oral prednisolone dose was tapered; consolidations did not worsen (Figure 1H). On day 52 after hospitalization, the patient was discharged because he had been administered intravenous imipenem‐cilastatin for approximately 1 month.

**TABLE 1:** Laboratory data at admission, the results of BALF test, and the results of sputum AFB test with drug susceptibility after bronchoscopy.

Blood examinations	Result	Blood examinations	Result	BALF	Result	Sputum	Result
Complete blood count		Total bilirubin (mg/dL)	0.6	Total cell count (/mL)	78,000	AFB smear	(+)
White blood cells (/μL)	17,700	Sodium (mmol/L)	135	Neutrophil (%)	11	AFB culture	*M. goodii*
Neutrophil (%)	98.0	Potassium (mmol/L)	4.6	Lymphocyte (%)	23	**Drug susceptibility of *M. goodii***	**MIC**
Monocyte (%)	2.0	Chloride (mmol/L)	96	Monocyte (%)	62	Imipenem‐cilastatin	<2
Lymphocyte (%)	0.0	C‐reactive protein (mg/dL)	9.91	Eosinophil (%)	4	Meropenem	<2
Haemoglobin (g/dL)	12.9	KL‐6 (U/mL)	656	CD4/8 ratio	5.55	Amikacin	<4
Platelets (×10^4^/μL)	34.3	Rheumatoid factor (IU/mL)	(−)	Bacterial culture	(−)	Clarithromycin	**32**
Blood chemistry		Anti‐CCP antibody (U/mL)	(−)	AFB culture	(−)	Doxycycline	<0.5
Aspartate aminotransferase (U/L)	21	Anti‐Nuclear antibody	(−)			Faropenem	4
Alkaline phosphatase (U/L)	18	Anti‐ARS antibody	(−)			Tobramycin	2
Lactate dehydrogenase (U/L)	184	Anti‐SS‐A antibody (U/mL)	(−)			Azithromycin	>128
Alkaline phosphatase (U/L)	80	Anti‐SS‐B antibody (U/mL)	(−)			Linezolid	<1
γ‐glutamyl transpeptidase (U/L)	19	Anti‐U1‐RNP antibody	(−)			Levofloxacin	<1
Total protein (g/dL)	6.4	Anti‐Scl70 antibody	(−)			Sitafloxacin	<0.25
Albumin (g/dL)	3.3	Anti‐centromere antibody	(−)			Moxifloxacin	<0.25
Urea nitrogen (mg/dL)	18	PR3‐ANCA	(−)			Sulfamethoxazole‐trimethoprim	<0.25
Creatinine (mg/dL)	0.71	MPO‐ANCA	(−)			Clofazimine	0.25

Abbreviations: AFB, acid‐fast bacilli; ANCA, antineutrophil cytoplasmic antibody; ARS, aminoacyl tRNA synthetase; BALF, bronchoalveolar lavage fluid; CCP, cyclic citrullinated peptide; CD, cluster of differentiation; DNA, deoxyribonucleic acid; KL‐6, Krebs von den Lungen‐6; *M. goodii*, *Mycobacterium goodii*; MIC, minimum inhibitory concentration; MPO, myeloperoxidase; PR3, proteinase3; RNP, ribonucleoprotein; SS, Sjogren's syndrome.

The regimen was changed to intravenous amikacin (10 mg/kg/day, twice weekly), oral ciprofloxacin (400 mg/day), and oral doxycycline (100 mg/day). Three months after antimycobacterial therapy initiation, three consecutive sputum cultures revealed negative results. Therefore, intravenous amikacin administration was discontinued. The prednisolone dose was gradually tapered; oral ciprofloxacin and doxycycline were continued for 11 months. Results on sputum cultures remained consistently negative; consolidations on chest radiography improved.

## DISCUSSION

This case report presents three notable clinical findings. First, *M. goodii* rarely can cause pulmonary disease. Second, *M. goodii* pulmonary disease can be treated with a multidrug regimen based on in vitro susceptibility test results. Third, *M. goodii* pulmonary disease can induce secondary OP.

*M. goodii*, a rare pathogen, causes pulmonary disease. A literature review reporting 10 cases of *M. goodii* pulmonary disease, including the present case, is presented in Table 2.^1^, ^4^, ^5^, ^6^, ^7^ In these cases, age distribution comprised all age groups (median age, 54.5 years; range, 15–80 years), with a male predominance (men:women, 7:3). Either productive or dry cough; fever; dyspnea; and chest pain have been reported. Thus, the symptoms were not specific to *M. goodii* pulmonary disease and were similar to those of other NTM pulmonary diseases.^19^ Regarding the cases of *M. goodii* pulmonary disease, lipoid pneumonia, achalasia, and a history of gastrectomy were reported as comorbidities in 3, 2, and 3 cases, respectively.^1^, ^4^, ^6^, ^7^ Chronic aspiration due to exposure to mineral oil or difficulty ingesting oily substances linked to oesophageal or swallowing disorders, subsequently resulting in lipoid pneumonia, have been identified as risk factors for pulmonary diseases caused by rapid growing NTM, including *M. goodii*.^1^, ^20^ Therefore, oesophageal and gastric anatomical or functional abnormalities may be risk factors for developing *M. goodii* pulmonary disease. Considering chest image findings of *M. goodii* pulmonary disease, consolidations were observed in four of 10 cases.^5^, ^6^, ^7^ NTM pulmonary disease related to achalasia commonly showed patchy bilateral consolidations resembling aspiration pneumonia.^21^ Thus, chest image findings of *M. goodii* pulmonary disease may be the features of *M. goodii* itself or involve patients' comorbidities.

**TABLE 2:** Cases of *Mycobacterium goodii* pulmonary disease.

Case	Author	Year	Age and Sex	Specimen in which *M. Goodii* detected	Symptoms	Chest image findings	Comorbidities	Treatment
1	Wallace et al.^4^	1988	58, M	Pleural fluid, lung biopsy	N/A	N/A	Lipoid pneumonia	N/A
2	Brown et al.^1^	1999	76, M	Lung biopsy	N/A	N/A	N/A	N/A
3			56, M	BALF	N/A	N/A	N/A	N/A
4			34, F	Lung biopsy	N/A	N/A	Lipoid pneumonia, prior gastrectomy	N/A
5			18, M	Lung biopsy	N/A	N/A	Lipoid pneumonia	N/A
6			53, M	Sputum	N/A	N/A	Achalasia	N/A
7	Buijtels et al.^5^	2005	66, M	Pleural fluid	Productive cough, chest pain	Pleural effusion, peribranchial pathology, and consolidation on the CXR	N/A	N/A
8	Martinez‐Gonzales et al.^6^	2011	15, F	Sputum	Productive cough, fever	Bilateral GGO with consolidations on the chest CT	Achalasia	12 months of oral ciprofloxacin and doxycycline
9	Waldron et al.^7^	2019	51, F	Lung biopsy	Dry cough, dyspnea, and chest pain	Consolidations on the chest CT	Prior gastrectomy	6 weeks of IV amikacin, IV meropenem, oral ciprofloxacin, and oral doxycycline Subsequently, 2 years of oral sulfamethoxazole‐trimethoprim and ciprofloxacin
Our case		2023	80, M	Sputum	Productive cough, fever	Consolidations on the chest CT	Prior gastrectomy	4 weeks of IV imipenem‐cilastatin, 3 months of IV amikacin, being continued oral doxycycline and oral ciprofloxacin

Abbreviations: BALF; bronchial alveolar lavage fluid; CT, computed tomography; CXR, chest radiograph; F, female; GGO, ground glass opacity, IV, intravenous; M, male; *M. goodii*, *Mycobacterium goodii*; N/A, not available.

Evidence regarding the regimen and treatment duration for *M. goodii* pulmonary disease remains insufficient. The official clinical practice guidelines recommend treating common NTM, such as *Mycobacterium avium complex* (MAC), with a multidrug regimen based on in vitro drug susceptibility test results.^22^ In regard to relatively uncommon NTM pulmonary diseases, a consensus statement recommends that the treatment regimen should be chosen based on in vitro drug susceptibility test results.^23^ Regarding both common and relatively uncommon NTM pulmonary diseases, the treatment duration should be at least 12 months after negative culture results. Furthermore, the duration of treatment of rapidly growing NTM pulmonary diseases should be divided into an initial phase of at least 1 month with >3 drugs containing at least one intravenous drug and a continuation phase of >2 oral drugs.^22^, ^23^ Treatment regimens for *M. goodii* pulmonary disease have been reported in two cases (cases 8 and 9, Table 2).^6^, ^7^ One patient was treated with two oral antibiotics.^6^ Another patient was treated with a multidrug regimen involving two intravenous antibiotics, in accordance with clinical guidelines and consensus statements for other rapidly growing NTM pulmonary diseases.^7^ In our case, a multidrug regimen was initiated. The treatment regimen resulted in negative culture results within 3 months. Thus, two‐phase treatment based on in vitro drug susceptibility test results may be effective in *M. goodii* pulmonary disease. However, the optimal regimen and treatment duration for *M. goodii* pulmonary disease remain unknown.

OP may be caused by *M. goodii* pulmonary disease. OP may be induced by NTM pulmonary disease as with other microorganism infections, or it may be one of the clinicopathological features of NTM pulmonary disease itself. Proinflammatory cytokines, such as tumour necrosis factor‐α and interleukin‐6, may be involved in OP development.^24^, ^25^, ^26^, ^27^ Conversely, MAC pulmonary disease may be involved in the release of tumour necrosis factor‐α and interleukin‐6.^28^, ^29^ A similar response during OP development may occur in patients with MAC pulmonary diseases. However, whether similar mechanisms occur in other NTM pulmonary diseases has not been verified. A literature review of previous cases of pathologically proven OP induced by NTM pulmonary disease is presented in Table 3. OP caused by NTM pulmonary disease was improved by antimycobacterial therapy with systemic corticosteroids and sometimes antimycobacterial therapy alone, except in resection cases.^7^, ^11^, ^12^, ^13^, ^14^, ^15^, ^16^, ^17^, ^18^ In the three cases, including the present case, administering antimycobacterial therapy alone showed insufficient therapeutic effect on OP caused by NTM pulmonary disease. The addition of systemic corticosteroids to the antimycobacterial therapy regimen resulted in improved pulmonary consolidations.^15^, ^17^ On the other hand, in three cases wherein only systemic corticosteroids were initially administered, pulmonary consolidations responded poorly to systemic corticosteroids. The addition of antimycobacterial agents to the systemic corticosteroids regimen resulted in improved pulmonary consolidations.^7^, ^12^, ^18^ Thus, OP caused by NTM pulmonary disease should be treated with antimycobacterial therapy; however, the addition of systemic corticosteroids is often likely to be effective. Similarly, in cases of OP caused by *M. goodii* pulmonary disease, administering antimycobacterial therapy and systemic corticosteroids may be effective.

**TABLE 3:** Cases of non‐tuberculous mycobacterium pulmonary disease accompany with pathologically proven OP.

Case	Author	Year	Age and Sex	Specimen in which OP was proven	Pathogen	Treatment for NTM pulmonary disease and secondary OP
1	Marchevsky et al.^11^	1982	31, F	SLB	MAC	N/A
2			75, F	SLB	MAC	N/A
3			79, M	SLB	MAC	N/A
4			60, F	SLB	MAC	N/A
5			68, M	SLB	*M. gordonae*	N/A
6			55, F	SLB	*M. fortuitum*	N/A
11	Hamada et al.^12^	2006	67, F	TBLB	*M. intracellulare*	Systemic corticosteroids only, subsequently systemic corticosteroids + antimycobacterial therapy
12	Jones et al.^13^	2009	58, M	TBLB	MAC	Systemic corticosteroids + antimycobacterial therapy
13	Starobin et al.^14^	2011	85, F	TBLB	*M. kansasii*	Systemic corticosteroids + antimycobacterial therapy
14	Nakahara et al.^15^	2015	66, F	SLB	*M. avium*	Resection
15			74, M	SLB	*M. avium*	Resection
16			65, M	TBLB	*M. avium*	Antimycobacterial therapy
17			73, M	TBLB	*M. avium*	Systemic corticosteroids + antimycobacterial therapy
18			66, F	TBLB	*M. abscessus*	Antimycobacterial therapy only, subsequently systemic corticosteroids + antimycobacterial therapy
19	Hong et al.^16^	2017	67, F	Percutaneous lung biopsy	*M. abscessus*	Systemic corticosteroids + antimycobacterial therapy
20	Watanabe et al.^17^	2019	59, M	TBLB	*M. abscessus*	Antimycobacterial therapy only, subsequently systemic corticosteroids + antimycobacterial therapy
21	Waldron et al.^7^	2019	51, F	SLB	*M. goodii*	Systemic corticosteroids only, subsequently systemic corticosteroids + antimycobacterial therapy
22	Fernandes et al.^18^	2021	64, F	Percutaneous lung biopsy	*M. avium*	Systemic corticosteroids only, subsequently systemic corticosteroids + antimycobacterial therapy
Our case		2023	80, M	TBLC	*M. goodii*	Systemic corticosteroids only, subsequently antimycobacterial therapy only, finally systemic corticosteroids + antimycobacterial therapy

Abbreviations: F, female; M, male; MAC, *Mycobacterium avium complex*; *M. abscessus*, *Mycobacterium abscessus*; *M. avium*, *Mycobacterium avium*; *M. fortuitum*, *Mycobacterium fortuitum*; *M. goodii*, *Mycobacterium goodii*; *M. gordonae*, *Mycobacterium gordonae*; *M. kansasii*, *Mycobacterium kansasii*; *M. intracellulare*, *Mycobacterium intracellulare*; NTM, non‐tuberculous mycobacterium; N/A, not available; OP, organizing pneumonia; SLB, surgical lung biopsy; TBLB, transbronchial lung biopsy; TBLC, transbronchial lung cryobiopsy.

*M. goodii* causes pulmonary disease that can be treated with a multidrug regimen based on in vitro drug susceptibility test results. *M. goodii* pulmonary diseases might induce dysregulated immune responses in the lungs such as OP. Systemic corticosteroids combined with antimycobacterial therapy might be effective against immune responses caused by *M. goodii* pulmonary disease.

## AUTHOR CONTRIBUTIONS

Yu Shionoya cared for the patient during hospitalization and wrote this manuscript, Hajime Kasai wrote and revised the manuscript, Reiya Kono helped writing the manuscript, Ryutaro Hirama cared for the patient during hospitalization and supervised this case report, Masayuki Ota made pathological diagnosis and provide pathological images, Akira Naito cared for the patient during hospitalization and supervised this case report, Mitsuhiro Abe cared for the patient during hospitalization and supervised this case report, Jun‐ichiro Ikeda supervised pathological diagnosis, Takeshi Kawasaki cared for the patient during hospitalization and supervised this case report, Takuji Suzuki supervised this case report.

## CONFLICT OF INTEREST STATEMENT

None declared.

## ETHICS STATEMENT

The authors declare that appropriate written informed consent was obtained for the publication of this manuscript and accompanying images.

## Data Availability

The data that support the findings of this study are available from the corresponding author upon reasonable request. The data are not publicly available due to privacy or ethical restrictions.
